# Supplementary material for: A Transcriptomic Meta-Analysis Shows Lipid Metabolism Dysregulation as an Early Pathological Mechanism in the Spinal Cord of SOD1 Mice
Source: Int J Mol Sci. 2021 Sep 2;22(17):9553. doi: 10.3390/ijms22179553 (PMC8431303; doi:10.3390/ijms22179553)
Supplement: Supplementary file 1 [file ijms-22-09553-s001.zip › Supplementary Files/Figura Supl.pdf]

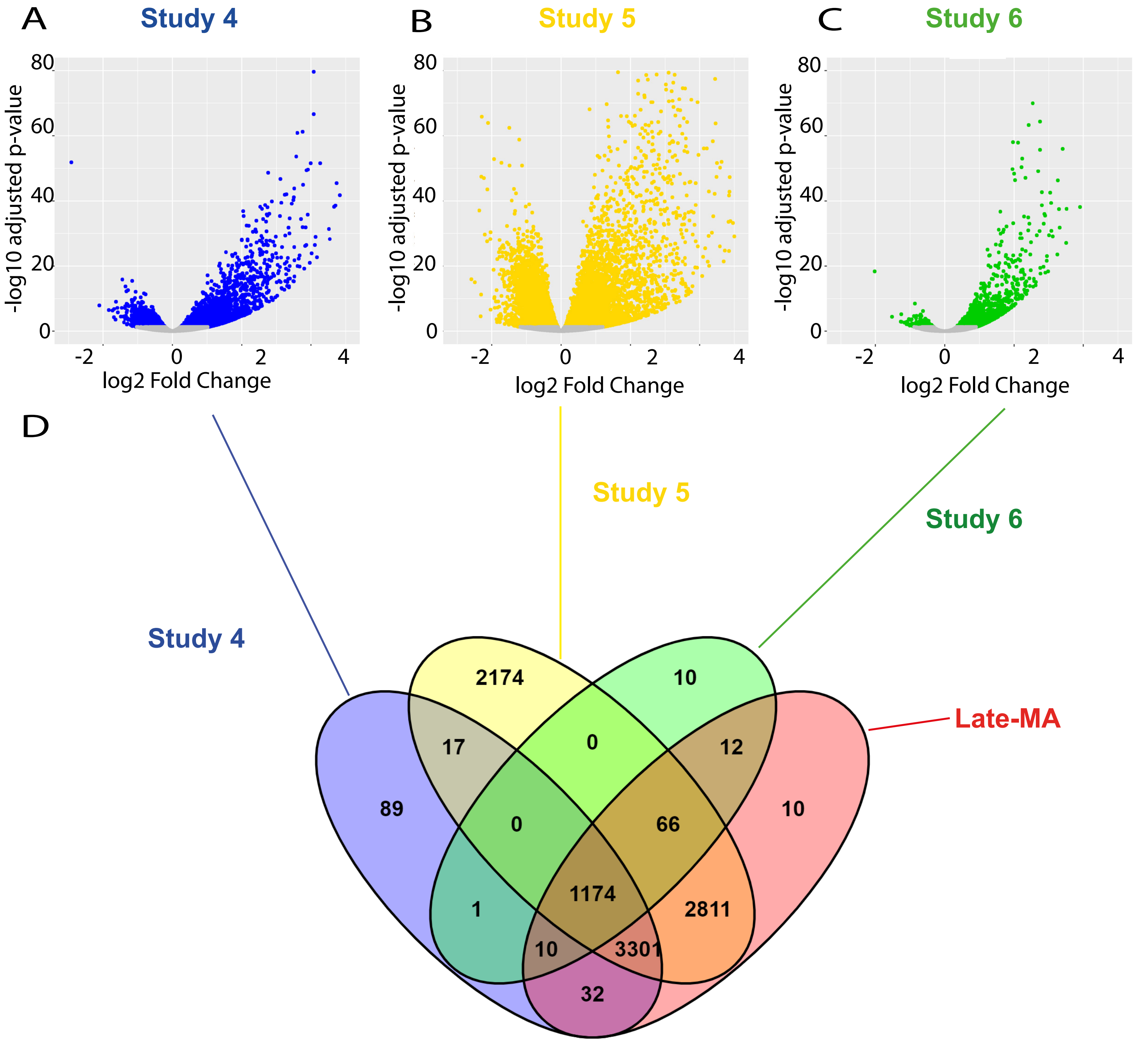

Figure S1. Meta-analysis of late-symptomatic RNA-seq studies. (**A-C**) Volcano-plots showing the dysregulated genes found on each of the three RNA-seq studies selected at late symptomatic . Blue, yellow and green dots indicates DEGs found in the study 4, 5 and 6 respectively. Grey dots indicate genes with no significant change. (**D**) A Venn diagram illustrating the overlapping DEGs across the three studies and the result of the meta-analysis (Late-MA). The meta-analysis (Late-MA) was performed using Fishers combined probability test where raw p-values were adjusted by the Benjamini–Hochberg false discovery rate (FDR) method and the adjusted p-values less than 0.01 were considered as statistically significant.

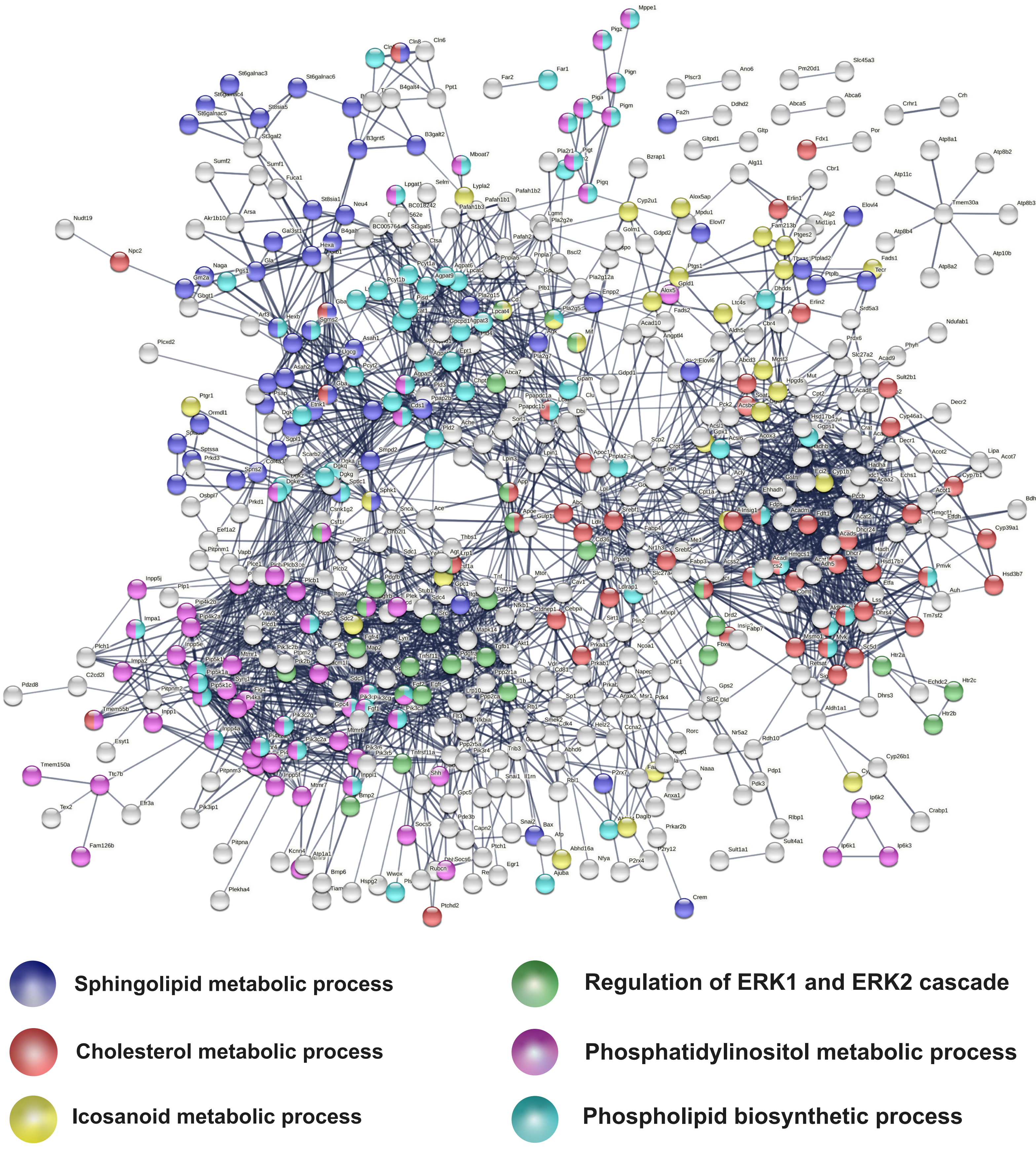

Figure S2. Protein-protein interaction network of lipid DEGs from late-symptomatic meta-analysis. The six relevant lipid GO terms are coloured-coded as follows: sphingolipid metabolic processes in blue, cholesterol metabolic processes in red, icosanoid metabolic processes in yellow, phospholipid biosynthetic processes in cyan, regulation of ERK1 and ERK2 cascade in green, and phosphatidylinositol in pink.

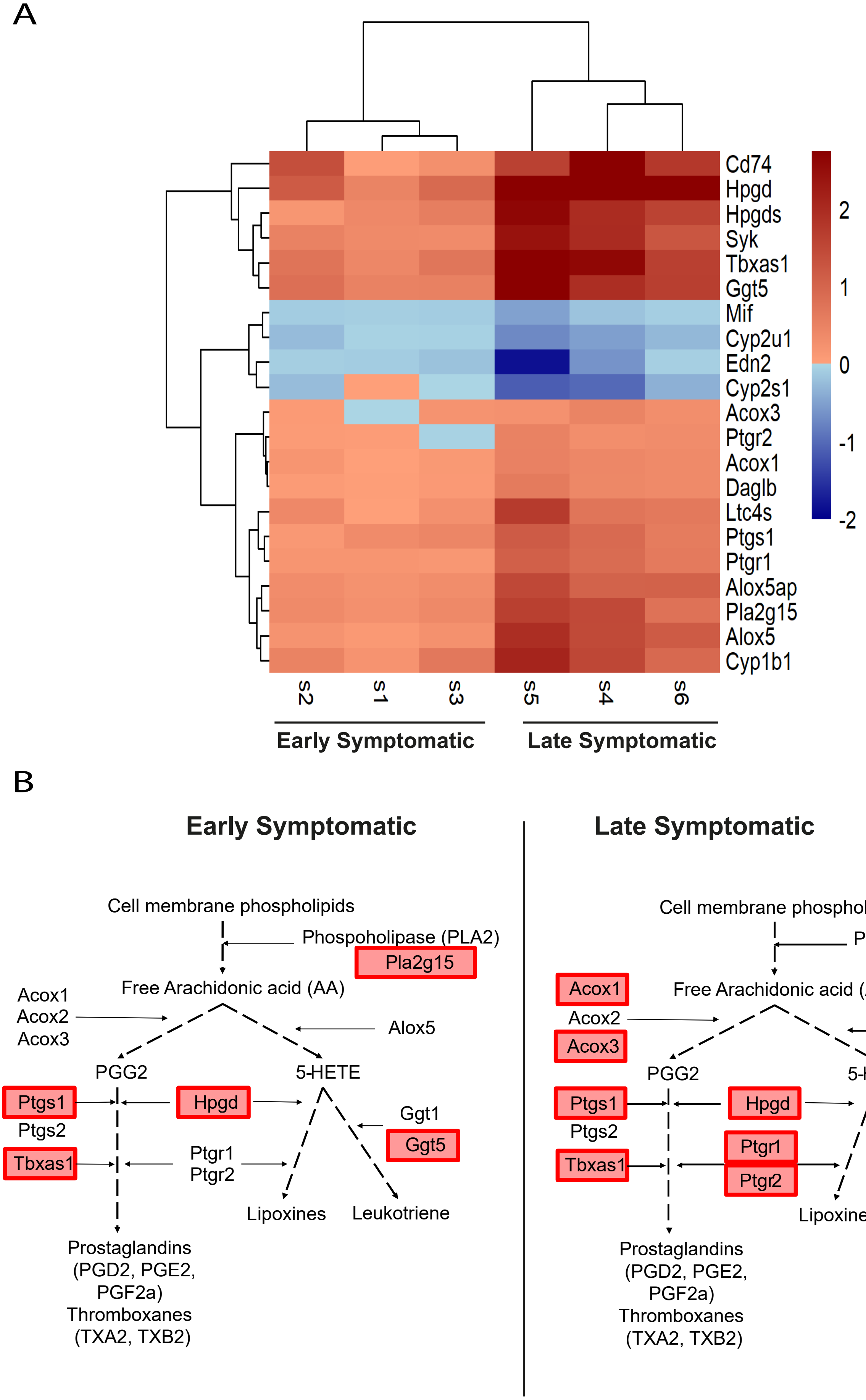

Figure S3. Comparative transcriptional alterations in icosanoids metabolic pathways in the spinal cord of SOD1 mice at early- and late-symptomatic disease stages. (A) The heatmap of the combination of the six transcriptomic studies (three early-symptomatic and three late-symptomatic) shows the expression level of the altered genes that participate in the icosanoids metabolic pathways. The heatmap colours indicate the expression levels by the log2-fold-change. Each column represents an individual RNA-seq study, and each row represents one gene. (B) The icosanoids biosynthesis pathways are represented, comparing the results from the Early-MA and the Late-MA. Coloured genes indicate that the gene was significantly altered in the meta-analysis. Blue indicates down-regulation and red indicates up-regulation.
